# Adaption and validation of Nijmegen continuity questionnaire to recognize the influencing factors of continuity of care for hypertensive patients in China

**DOI:** 10.1186/s12913-019-3915-6

**Published:** 2019-01-29

**Authors:** Chen Qiu, Shixiang Chen, Ying Yao, Yue Zhao, Yi Xin, Xiaoying Zang

**Affiliations:** 1 School of Nursing, Tianjin Medical University, Qixiangtai Road, Heping District, Tianjin, China; 2 Department of Emergency, Tianjin Medical University General Hospital, Tianjin, China; 3 Department of Cardiology, Second Affiliated Hospital of Tianjin University of Traditional Chinese Medicine, Tianjin, China

**Keywords:** Continuity of care, Hypertension, Chronic diseases, Reliability, Validity

## Abstract

**Background:**

Continuity of care (COC) has become a primary point of concern for care providers in both developed and developing countries, which is regarded as the “cornerstone of care” and an “essential element” of good health care. A robust and proper instrument is of necessity to identify problems and evaluate intervention aimed at improving continuity of care. This study aimed to adapt Nijmegen continuity questionnaire (NCQ) into a Chinese version (NCQ-C) and to delineate the status of COC as well as explore its influencing factors for hypertensive patients in China.

**Methods:**

A forward-back-translation procedure was adopted for the determination of the adaption of NCQ. Then a total of 448 patients completed questionnaires and 24-h ambulatory blood pressure monitoring (ABPM). Proper indexes were calculated to test the reliability and validity of NCQ-C. Logistic analysis were used to detect the influencing factors of COC.

**Results:**

The NCQ-C had excellent intraclass correlation coefficient of 0.855 and internal consistency of seven dimensions varied from 0.907 to 0.944. The item-content validity index ranged from 0.71 to 1.00. For construct validity, seven-factor structure was confirmed as original questionnaire and all the fit indices indicated acceptable levels. Gender, education level, medical insurance and frequency of family visits, blood pressure level, depression status as well as general health perception were demonstrated to be statistically related to COC.

**Conclusions:**

In addition, all the parameters of ABPM were negatively significant with COC. The NCQ-C has shown acceptable level of reliability and validity. The related factors of COC should arouse care providers’ attention.

## Background

The ranking of chronic diseases reached the top position of spectrum of diseases since twenty-first century. According to the report on the global status of chronic diseases, nearly two-thirds of death all over the world could be explained by chronic diseases [1]. The numbers of patients with one or more chronic diseases are increasing and these patients are prone to go through referral among different medical settings and communicate with various health care providers [2], which lead to the potential risk of “fragmentation of health care” [3]. Therefore, continuity of care (COC) gradually became a primary point of concern for care providers in both developed and developing countries. COC is regarded as the “cornerstone of care” and an “essential element” of good health care [4, 5]. A substantial of evidence bodies have indicated that greater COC was related to lower readmission rate [6], higher quality of life [7] and more satisfaction [8].

According to literature review, COC first appeared in the 1950s [9–11]. It is a complex concept which has changed over time due to contextual factors. At different times, researchers in various organizations emphasized COC from different aspects [10]. Around mid-1970s, COC was thought to be the synonym of building relationships with the same health care providers [12, 13]. Several measurement instruments of COC, such as Usual Provider of Continuity (UPC) Index [7], the sequential continuity index (SCN) [14] were developed, all of which pursued largely from a service-orientated, clinician-centered perspective [15]. From the 1990s on, with much more in-depth and integrated understanding, providers realized that research on COC would benefit from a much stronger focus on the patients’ perspective [16] and tended to endorse COC a multi-facial definition. Nowadays, COC was defined from patients’ viewpoint as “the patients’ experience of a coordinated and smooth progression of care” [10] and was considered as a multi-faced model including not only personal or relational continuity but also informational continuity and team/cross-boundary continuity requiring communication and collaboration between care providers [17, 18]. But for some patients, informational continuity and team/cross-boundary continuity were hard to distinguish [19].

To optimize management process of hypertensive patients and achieved optimal management effectiveness, China passed the notice on the pilot work of hierarchical diagnosis and treatment for patients with hypertension in 2015 [20]. It depicted the process of hierarchical diagnosis and treatment which could be achieved by the collaboration between primary health care and high-lever hospitals through bi-directional referral system. Specifically, patients with hypertension in China were managed in cooperation among a panel of care providers including general practitioners, nurses in primary care settings as well as specialists and nurses in higher level hospitals, and all these health care providers are assigned with definite division of labor [21, 22]. General practitioners who work as gatekeepers on communities or clinics basis, guarantee daily management for hypertensive patients [23]. They play a prominent role to provide basic prescription and referee emergency patients to higher level hospitals. Nurses in primary care settings assisted general practitioners to perform routine family visits and patient education, counseling program [24], which have been proved to be effective to improve lifestyle modification and antihypertensive medications. Specialists and nurses in higher level hospital provide necessary instructions when hypertensive patients with deteriorating status were referred to seek further treatment [25]. The entire service process emphasize the necessity of collaboration among care providers and information handover within and between primary care and hospitals, and personal continuity and commitment is crucial to build trust and promote partnerships with all health care providers involved in [26], which further demand the need for higher level of COC. Measurement of COC by robust and convenient instruments would allow us to identify problems and evaluate intervention aimed at improving COC. However, there was a blank for reliable and specific instruments concerning COC for hypertensive patients in China. Nijmegen continuity questionnaire (NCQ) is a general questionnaire [19] and it is the only questionnaire that has been tested in both primary and secondary care [27]. NCQ can not only measure COC from a macro scope to evaluate the entire quality of the medical service, but also a micro scope to assess single (respective) COC of primary care and hospital [28], which suits medical system in China to a large extent. The purpose of this study was to adapt NCQ into a Chinese version (NCQ-C) and to describe the status of COC as well as explore the influencing factors of COC for hypertensive patients in China.

## Methods

### Translation and adaption

The original questionnaire was developed by Annemarie in Netherlands [19]. With the permission of original author, a forward-back-translation procedure was adopted as recommended in a guideline by Beaton et al. [29]. Two authors, a post graduate student majoring in nursing and a professor who specializes in continuity of care, translated it into Chinese based on the English version of NCQ with the help of healthcare professionals and linguists in China. Then a native English-speaking translator performed the process of back-translation. After a pilot study among 30 participants and discussion in the research group, the consensus version was developed. After adaptation, NCQ-C are applicable in China. The instrument consists of 28 items that distributes into three subscales:

- Personal continuity: care provider knows me (5 items each for general practitioner in primary care setting and specialist in hospital).
- Personal continuity: care provider shows commitment (3 items each for general practitioner in primary care setting and specialist in hospital).
- Team/cross-boundary continuity (4 items each for collaboration between care providers within primary care setting, within the hospital/outpatient department and between the primary and hospital care providers).

Patients were instructed to fill the questionnaire selectively according to their doctoring behavior in the past 12 months. The items on continuity were rated according to a five-point Likert scale ranging from 1 (strongly disagree) to 5 (strongly agree), with an additional option to choose “?” (“I do not know”). Principally, the model had three subscales, but the subscale of “personal continuity-healthcare provider knows me” and “personal continuity- healthcare provider shows commitment” were used for both general practitioner and hospital specialist, and the team/cross boundary subscale was applied to three team contexts: in primary care setting, in hospital, and between primary care setting and hospital, giving a seven-factor model, which had been tested by the Norwegian version of NCQ [30]. Compared to the three-factor model, we believed that the seven-factor model addressing different levels of care settings were more suitable to be extensively used under the hierarchal treatment system in China.

### Participants and data collection

A multi-center study was conducted in three tertiary hospitals in Tianjin, China. The three hospitals distributed in different geographical regions of Tianjin and covered large parts of this City with abundant outpatients. Between September 2017 and February 2018, 448 patients with hypertension were recruited into our research, and these patients were referred from nearby community healthcare centers to hypertension outpatients to undergo routine physical examination or further treatment. Patients were eligible if they met the following criteria: (1) being clearly diagnosed with primary hypertension for at least 1 years, according to the Chinese guideline for the management of hypertension in 2010: average clinical systolic blood pressure (SBP) more than 140 mmHg or (and) diastolic blood pressure (DBP) more than 90 mmHg [31]; (2) receiving anti-hypertensive therapies for at least 3 months; (3) aging more than 18 years old; (4) able to understand and write Chinese; (5) willing to participate the research and accept the examination of ambulatory blood pressure monitoring (ABPM). Exclusion criteria included active and severe infection, impaired cognitive abilities. This study was approved by the Research Ethics Committees of Tianjin Medical University.

Procedure: First off, all participants were told the purpose of the research and provided with a written informed consent. After receiving their consent, they were asked to complete a package of measures: demographic and clinical information, NCQ-C, Duke Activity Status Index (DASI), Self-rating Depression Scale (SDS) and visual analog scale (VAS) of European Quality of Life-5 Dimensions (EQ-5D). The self-made demographic and clinical questionnaire included the items of age, gender, marital status (married or other), income, education level (senior high school or lower, college or higher), employment (employed and unemployed), medical insurance, comorbidities and duration of hypertension, body mass index (BMI) and the frequency of family visits. The income was categorized into 4 classes, < 4000, 4001–6000, > 6000 RMB according to the consumption expenditure and income per capita announced by Tianjin Bureau of Statistics. Based on the guideline of prevention and treatment of hypertension in primary care, primary care providers must carry out family visit at least once every 3 months, so the frequency of family visit was set as dichotomous variable with the option of less than 4 times per year or more. Patients’ comorbidities were tested by Charlson comorbidity index [32], which was broadly used in experimental and clinical researches.

DASI is a 12-item scale with acceptable reliability and validity, and the Cronbach’s α is 0.704 [33]. Each item has two response options (‘yes’ or ‘no’), and the total scores range from 0 to 58.2, with higher scores indicating better physical functional capacity.

SDS is a widely-used scale with 20 items. Each item is rated from 1 to 4. The total score range from 1 to 80, wherein the higher scores reflect more severe depression status. The Chinese version of SDS is confirmed to be valid [34].

VAS of EQ-5D allowed the participants to rate their current health status on a range from 0 (worst imaginable health status) to 100 (best imaginable health status). It has been extensively used for its convenience and brevity and the Cronbach’s α is 0.75 [35].

After finishing those questionnaires, non-invasive ABPM was performed with automatic device (Welch Allyn. Inc. ABPM 6100) which measured BP on the upper left arm [36]. The monitoring lasted for 1 day from 10:00 am to 10:00 am the following day. With fixed time of the inflation and deflation of the cuff, the readings about corresponding parameters were recorded every 30 min during the diurnal period (07:00 to 23:00) and every 60 min during the nocturnal period (23:00 to 08:00). During the monitoring period, the patients kept their daily activities with unchanged lifestyles, but they should keep still and put their right upper limbs in the proper position when the cuff started to inflate. Each subject needed to provide at least 32 readings. Patients’ blood pressure level was dichotomized as normal or abnormal based on both their 24-h systolic and diastolic blood pressure. Their blood pressure pattern was judged by nocturnal reduction of SBP. In this research, the patterns of BP would be divided into 2 groups: normal pattern: dipper (≥10%, but < 20%), abnormal pattern: non-dipper (> 0%,but < 10%), reverse-dipper (no decline at night, that is < 0%) and super-dipper (> = 20%) [31]. In addition, 35 patients were randomly selected to complete the questionnaires 2 weeks later through interviewing by telephone, and the test-retest reliability was examined.

### Statistical analysis

Data management and analysis was performed using SPSS20.0 software. Continuous variables with normal distribution were expressed as mean ± standard deviation (x ± s), non-normal variables were presented as median (interquartile range) and categorical variables were presented as number and percentage.

### Reliability

Test-retest reliability was calculated by two-way random effects of average measure intraclass correlation coefficient (ICC) for absolute agreement between two tests [37] and ICC > 0.75 indicates acceptable reproducibility [38]. Internal consistency of the scale was examined by calculating the Cronbach’s alpha of every parts, which was considered satisfactory between 0.70 and 0.95 [39].

### Validity

The content validity and construct validity was adopted to evaluate whether the questionnaire had proper validity. We invited 3 experts who majored in continuity of care, chronic diseases care and primary care, respectively and two doctors, two nurses from different community healthcare centers and hospitals to perform the evaluation of content validity index (CVI). A four-point ordinal rating scale was used, where 4 = strong relevant, 3 = very relevant, 2 = weak relevant, and 1 = not relevant. Both item-content validity index (I-CVI) and scale-level content validity index /average agreement (S-CVI/Ave) were adopted to quantitative evaluate the scale-level content validity [40]. I-CVI was calculated as the number of experts giving a rating of either “strong relevant” or “very relevant”, divided by the number of experts. S-CVI/Ave was calculated by taking the average of the I- CVIs. A scale with an I-CVI value > 0.78 is considered perfect and an S-CVI/Ave is anticipated to achieve 0.90 [40, 41]. For construct validity, we performed confirmatory factor analysis (CFA) to detect seven-factor structure similarly to the original questionnaire. Factors were allowed to correlate with each other. The sufficiency of the construct was evaluated using goodness of-fit statistics [42] including χ2/df, Goodness of Fit Index (GFI), Comparative Fit Index (CFI), Tacker-Lewis Index (TLI) and Standardized Root Mean Square Residual (SRMR). The values of χ2/df ranging from 1 to 3, GFI > 0.90, CFI > 0.90, TLI > 0.95, SRMR < 0.05, RMSEA < 0.08 were regarded as acceptable model fit [43].

### Influencing factors of COC

Mean score of COC was seen as a cut-off and binary logistic regression (“0” = upper the mean, “1” = below the mean) was performed to determine the influencing factors of COC. *P* values < 0.05 was considered statistically significant.

## Results

### Demographic and clinical characteristics of participants

Participants’ characteristics: Totaling 448 participants were recruited into the research, all of whom performed doctoring behaviors both in primary care centers and hospital. The sociodemographic and clinical information about participants were presented in Table 1. A large proportion of participants were female with an average age of 61.7 years old. Over half (75.0%) of participants were with medical insurance and most of them (72.8%) were unemployed.

**Table 1:** Demographic and clinical characteristics of participants (*N* = 448)

Characteristics	Number	Percent	Mean (SD)/Median(Q1-Q2)	Minimum-Maximum
Age			61.7 (12.3)	18–86
Gender				
Male	191	42.6		
Female	257	57.4		
Marital status				
Married	420	93.8		
Other	28	6.3		
Income				
≤4000	157	35.0		
4001–6000	224	50.0		
> 6000	67	15.0		
Education level				
Senior high school or lower	332	74.1		
College or higher	116	25.9		
Employment				
Employed	110	27.2		
Unemployed	326	72.8		
BMI			25.61 ± 3.70	16.16–25.61
Charlson Index			2 (1–3)	0–11
Duration of hypertension			5 (3–12)	1–45
Blood Pressure Level				
Normal	237	52.9		
Abnormal	211	47.1		
Blood Pressure Pattern				
Normal	114	25.4		
Abnormal	334	74.6		
Physical Activity			34.75 ± 9.52	14–42
Mental Function			39.53 ± 14.97	10–68
General Health Perception			73.56 ± 11.17	30–90
Medical insurance				
Yes	338	75.0		
No	110	25.0		
The frequency of family visit				
Less than 4 times per year	248	55.4		
More	200	44.6		

### Reliability

The ICC presented as the correlation between pretest and post-test of NCQ-C was 0.855 (CI: 0.736–0.933). The internal consistency of seven dimensions ranged from 0.907 to 0.944.

### Validity

With regard to the validity analysis, the I-CVC of the NCQ-C ranged from 0.71 to 1.00 and the S-CVI/Ave was 0.96. Except the item 5 (This care provider always remembers what he/she did during my last visit(s)), other items of this scale had I-CVI values more than 0.78. For item 5, the experts suggested to change “what I did” into explicit disease-related behaviors. Table 2 depicted experts’ ratings and CVI calculation. The results of construct validity revealed the same items distribution as the original questionnaire. Based on the analysis of CFA (Table 3), items loaded on seven factors with all factor loadings ranged from 0.946 to 0.999, and the correlations between seven factors ranged from 0.237–0.781. All the fit indices reached acceptable level.

**Table 2:** Experts’ Ratings and CVI Calculation (*N* = 7)

Item	Experts’ rating							Number of Three or Four Items	I-CVI	Evaluation
	A	B	C	D	E	F	G			
1	4	4	3	4	2	4	4	6	0.86	Excellent
2	4	4	4	4	4	4	4	7	1	Excellent
3	4	3	4	3	4	4	4	7	1	Excellent
4	4	4	4	4	4	4	4	7	1	Excellent
5	4	3	4	2	2	4	4	5	0.71	Good
6	4	4	4	4	4	4	4	7	1	Excellent
7	4	4	4	4	4	4	4	7	1	Excellent
8	4	4	4	4	3	4	4	7	1	Excellent
9	4	4	4	4	3	4	4	7	1	Excellent
10	4	4	4	4	4	4	4	7	1	Excellent
11	4	4	4	4	4	4	4	7	1	Excellent
12	4	4	3	4	4	4	4	7	1	Excellent

**Table 3:** Results of confirmatory factor analysis of the NCQ-C

Item	Factor loading	Squared correlations	Standard Error of variance
Personal Continuity: general practitioner knows me			
1 I know this care provider very well.	0.962	0.925	0.075
2 This care provider knows my medical history very well.	0.976	0.953	0.047
3 This care provider always remembers what he/she did during my last visit(s).	0.968	0.937	0.063
4 This care provider knows my family circumstances very well.	0.957	0.916	0.084
5 This care provider knows what I do in my day-to-day life very well.	0.941	0.885	0.115
Personal Continuity: general practitioner shows commitment			
1 This care provider contacts me when necessary without me having to ask him/her to do so.	0.996	0.992	0.008
2 This care provider knows very well what I think is important when it comes to my care.	0.984	0.968	0.032
3 This care provider maintains enough contact with me when I am seen by other care providers.	0.976	0.953	0.047
Team/Cross-boundary Continuity related to Primary Care Providers			
1 These primary care providers pass on information to each other very well.	0.996	0.992	0.008
2 These primary care providers work together very well.	0.985	0.970	0.030
3 The primary care given by these care providers is well-connected.	0.978	0.956	0.044
4 These primary care providers always know very well what the other care providers have done.	0.990	0.980	0.020
Personal Continuity: Specialist knows me			
1 I know this care provider very well.	0.911	0.830	0.170
2 This care provider knows my medical history very well.	0.896	0.803	0.197
3 This care provider always remembers what he/she did during my last visit(s).	0.925	0.856	0.144
4 This care provider knows my family circumstances very well.	0.779	0.607	0.393
5 This care provider knows what I do in my day-to-day life very well.	0.793	0.629	0.371
Personal Continuity: Specialist shows commitment			
1 This care providers contacts me when necessary without me having to ask him/her to do so.	0.998	0.996	0.004
2 This care providers knows very well what I think is important when it comes to my care.	0.991	0.982	0.018
3 This care providers maintains enough contact with me when I am seen by other care providers.	0.967	0.935	0.065
Team/Cross-boundary Continuity related to Hospital care providers			
1 These hospital care providers pass on information to each other very well.	0.969	0.939	0.061
2 These hospital care providers work together very well.	0.925	0.856	0.144
3 The hospital care given by these care providers is well-connected.	0.935	0.874	0.126
4 These care providers always know very well what the other care providers have done.	0.960	0.922	0.078
Team/Cross-boundary Continuity between primary and hospital care providers			
1 These care providers pass on information to each other very well.	0.967	0.935	0.065
2 These care providers work together very well.	0.951	0.904	0.096
3 The care given by these care providers is well-connected.	0.934	0.872	0.128
4 These care providers always know very well what the other care providers have done.	0.940	0.884	0.116
Model fit			
Chi-square / DF	2.656		
GFI	0.900		
CFI	0.982		
TLI	0.977		
RMSEA	0.061		
SRMR	0.005		

### Influencing factors of COC

The current status of COC among hypertensive patients was depicted in Table 4. In general, the scores of seven pacts of COC were at middle level. As showed in Table 5, when variables were tested by binary logistic regression analysis, gender, medical insurance, education level, the frequency of family visits, blood pressure level and depression status as well as general health perception of patients were proved to be influencing factors of COC. The specific tendency presented as: participants who were male (OR:1.513,CI:2.87–7.19), without medical insurance (OR:16.63,CI:6.25–44.21), with lower education level (OR:9.78,CI:3.44–27.91), underwent family visits less than 3 times per year (OR:14.09,CI:8.18–24.29), presented with abnormal blood pressure (OR:3.29,CI:2.21–4.89), with severe depression status (OR:1.15,CI:1.03–1.06) had high odds to have poor COC, but patients’ good general health perception (OR:0.908,CI:0.36–0.94) could increase their odds to get high level of COC.

**Table 4:** The score of seven components of NCQ-C (*N* = 448)

Part	Maximum Score	Actual Score	The Standard Score
Personal Continuity: general practitioner knows me	25	18.34 ± 4.49	73.37 ± 17.97
Personal Continuity: general practitioner shows commitment	15	8.33 ± 3.56	55.54 ± 23.74
Team/Cross-boundary Continuity related to Primary Care Providers	20	14.09 ± 3.73	70.45 ± 18.65
Personal Continuity: Specialist knows me	25	15.51 ± 3.30	62.05 ± 13.18
Personal Continuity: Specialist shows commitment	15	5.82 ± 1.94	38.84 ± 12.95
Team/Cross-boundary Continuity related to Hospital care providers	20	14.10 ± 3.26	70.49 ± 16.29
Team/Cross-boundary Continuity between primary and hospital care providers	20	13.37 ± 3.45	66.86 ± 17.26

**Table 5:** Results of logistic analysis of continuity of care

Variable (Reference)	B	Standard error	*P* value	OR (95% CI)
Gender (Female)	1.513	0.459	0.001	4.54 (2.87–7.19)
Education level (College or higher)	2.282	1.047	0.029	9.78 (3.44–27.91)
Medical insurance (Yes)	2.811	0.978	< 0.001	16.63 (6.25–44.21)
Family visit frequency (More)	2.646	0.544	< 0.001	14.09 (8.18–24.29)
Blood Pressure Level (Normal)	1.191	0.397	0.003	3.29 (2.21–4.89)
Depression Status	0.043	0.015	0.005	1.15 (1.03–1.06)
General Health Perception	−0.096	0.036	0.007	0.908 (0.36–0.94)

## Discussion

COC is an important aspect of patient care, which comprises continued and consistent care coupled with effective information exchange [44, 45]. Hypertension is a kind of chronic disease that threatens people by target organ damage and comorbidities in the long term [46]. In China, with the treatment concept shifting from solely lowing blood pressure to comprehensive prevention and treatment [21, 47], hypertensive patients were more likely to undergo transferring between different care settings and be treated under the collaboration among care providers. A robust and convenient instrument about COC for patients with hypertension is crucial to learn the current context and find potential factors for further intervention.

The results of reliability and validity were comparable to the previous studies using NCQ, which indicated that the instrument can be used to provide reliable results in other research in the future. The ICC of NCQ-C was 0.855, reflecting it had an excellent test-retest reliability and the internal consistency of NCQ-C was acceptable as well with the Cronbach’s alpha coefficient ranged from 0.907 to 0.944 for seven dimensions. In terms of content validity, 0.78 was regarded as cut-off value for either removing or retaining an item. Item 5 had I-CVI values less than 0.78, which suggested that further modification is needed in the future research. According to the results of CFA, the seven-factor structure of previous questionnaire was also suitable for NCQ-C with all fit indices reached an acceptable level.

In our research, we found several influencing factors of COC for hypertensive patients, and these factors can be divided into three groups: patients’ demographic factors, health status and factors related to care providers. Of the demographic factors examined, hypertensive patients who were female, with higher education level and medical insurance were inclined to get higher score of COC. The results were consistent to the previous research and can be reasonable interpreted. Compared to their counterparts, female patients showed high adherence to the treatment suggestion, which could boost the enthusiasm of their care providers in return [48]. Besides, high health literacy, as an advantage of well-educated patients [49, 50] could assist them to make most of medical resources [51, 52]. As for hypertensive patients with medical insurance in China, except for less burden of medical fee, these patients were registered in the primary care setting under the background of medical combination [53, 54], through which patients were more likely to contact with fixed care providers and care providers could achieve intimate collaboration from each other to ensure the same treatment target for the same hypertensive patients. Of factors related to patients’ health status, blood pressure level, depression status as well as patients’ general health perception were associated to COC, which was supported by the evidence that psychiatric comorbidities [55] as well as prior hospitalizations increased patients’ risk for poor COC [56]. In addition, health status was the fundamental index for patients to judge providers’ rating. Patients with good health state tended to show great appreciation and confidence in their health care providers, further leading to high level of continuity [57, 58]. It is noteworthy that hypertensive patients’ COC was more closely related to their mental disorders than physical liability, which hinted health care providers to emphasize both physical, psychological aspects in the treatment of hypertension. For factors related to care providers, the frequency of family visit played a crucial role for COC. The result indicated that care providers can perform increased frequency of family visit to achieve high level of COC for all patients. The assessment in family visit could serve as a media to achieve information sharing among care providers in different settings by case management system.

### Limitation

There were still some limitations for the research. Firstly, this was a cross-sectional study and a longitudinal research will contribute to test the sensitivity of the NCQ-C in the future. Second, in this research, all the participants were solely recruited from hospital care settings. On the basis of existed research, the questionnaire also applied to hypertensive patients recruited from primary care settings. Further study is needed in the future. Third, the participants were considered to be representative of Chinese patients with hypertension who live in Tianjin city and they may not be generalized to the overall population in China; a future study should recruit more participants from different regions in China.

## Conclusion

The Chinese version of NCQ has shown acceptable level of reliability and validity and can be used in the future research. Given the significant role of gender, education level, medical insurance and frequency of family visits for COC, care providers should emphasize the spectacular characteristics of patients and provide individual intervention in order to achieve optimal BP level.

## Abbreviations

ABPM: Ambulatory blood pressure monitoring
CFA: Confirmatory factor analysis
CFI: Comparative Fit Index
COC: Continuity of care
CVI: Content validity index
DASI: Duke Activity Status Index
DBP: Diastolic blood pressure
EQ-5D: European Quality of Life-5 Dimensions
GFI: Goodness of Fit Index
ICC: Intraclass correlation coefficient
I-CVI: Item-content validity index
NCQ: Nijmegen continuity questionnaire
SBP: Systolic blood pressure
SCN: Sequential continuity index
S-CVI/Ave: Scale-level content validity index /average agreement
SDS: Self-rating Depression Scale
SRMR: Standardized Root Mean Square Residual
TLI: Tacker-Lewis Index
UPC: Usual Provider of Continuity
VAS: Visual Analog Scale
